# A Tremella-Like Nanostructure of Silicon@void@graphene-Like Nanosheets Composite as an Anode for Lithium-Ion Batteries

**DOI:** 10.1186/s11671-016-1414-9

**Published:** 2016-04-16

**Authors:** Hongwei Mi, Fang Li, Shuxian Xu, Ziang Li, Xiaoyan Chai, Chuanxin He, Yongliang Li, Jianhong Liu

**Affiliations:** Shenzhen Key Laboratory of Functional Polymer, College of Chemistry and Environmental Engineering, Shenzhen University, Shenzhen, Guangdong 518060 People’s Republic of China

**Keywords:** Silicon nanoparticles, N-doped graphene-like nanosheets, Tremella-like, Lithium-ion batteries

## Abstract

Graphene coating is receiving discernable attention to overcome the significant challenges associated with large volume changes and poor conductivity of silicon nanoparticles as anodes for lithium-ion batteries. In this work, a tremella-like nanostructure of silicon@void@graphene-like nanosheets (Si@void@G) composite was successfully synthesized and employed as a high-performance anode material with high capacity, cycling stability, and rate capacity. The Si nanoparticles were first coated with a sacrificial SiO_2_ layer; then, the nitrogen-doped (N-doped) graphene-like nanosheets were formed on the surface of Si@SiO_2_ through a one-step carbon-thermal method, and the SiO_2_ layer was removed subsequently to obtain the Si@void@G composite. The performance improvement is mainly attributed to the good conductivity of N-doped graphene-like nanosheets and the unique design of tremella nanostructure, which provides a void space to allow for the Si nanoparticles expanding upon lithiation. The resulting electrode delivers a capacity of 1497.3 mAh g^−1^ at the current density of 0.2 A g^−1^ after 100 cycles.

## Background

To meet the further demands driven by the rapid development of portable electronics hybrid and electric vehicles, novel anode materials with higher energy density, low-cost, and long cycle life for lithium-ion batteries (LIBs) are of great interest [1–3]. Silicon has been recognized as one of the most promising and appealing anode materials, owing to the high natural abundance, low discharge potential, and especially its high theoretical specific capacity (4200 mAh g^−1^) which is ten times greater than that of a traditional graphite (~372 mAh g^−1^) [4, 5]. Unfortunately, Si suffers from the low conductivity and the severe volume fluctuation during the Li^+^ insertion/extraction, which can fracture the materials and lead to fast capacity fading. Moreover, the thickness of the insulating solid electrolyte interphase (SEI) film increases upon charge/discharge process, further degrading the capacity and cycling stability of Si electrode [6, 7]. These shortages cause much difficulty in the development of the silicon-based materials as commercial anode materials.

Many approaches have been developed to mitigate the above–mentioned challenges, including to decrease the Si particle sizes [8–13] and fabricate hollow or porous structure to confine volume expansion [14–18]. Tao et al. [17] prepared hollow core-shell structured Si/C nanocomposites to adapt for the large volume change. In addition, many Si-based materials were modified by new binder or conductive polymer [19–21], carbonaceous materials, such as amorphous carbon [22, 23], carbon nanotubes [24], graphite [25, 26], and graphene [27–32]. Graphene has been applied in LIBs in recent years, mainly due to its outstanding flexibility, electrical conductivity, and excellent mechanical strength [33–36]. For example, Wu et al. [29] synthesized the three-dimensional (3D) interconnected network of graphene-wrapped porous silicon spheres, and this electrode delivered a high reversible capacity of 1299.6 mAh g^−1^ after 20 cycles, exhibiting markedly enhanced performance compared with bare Si spheres.

Taking advantages of which offered by both hollow structure and graphene, we herein design a tremella-like nanostructure of silicon@void@graphene-like nanosheets (Si@void@G) composite as an anode for LIBs. The tremella-like structure with an internal void space can accommodate the large volumetric expansion of Si during lithiation. Moreover, nitrogen-doped (N-doped) graphene-like nanosheets can increase the electronic conductivity of the electrode. As an anode material for LIBs, the Si@void@G electrode delivers a reversible capacity of 1497.3 mAh g^−1^ at the current density of 0.2 A g^−1^ after 100 cycles, with the initial coulombic efficiency of 73.8 %, which exhibits significantly improved electrochemical performance than bare Si and silicon@graphene (Si@G) composite.

## Methods

### Materials and Preparation

Silicon nanoparticles (100 nm, Shanghai ST-NANO Science & Technology Co. Ltd., People’s Republic of China) were firstly dispersed into 400 mL of deionized alcohol-water (3:1 by volume) solution by sonication at room temperature for an hour. Then, 10 mL ammonia was added into this solution. After 5 min, tetraethoxysilane (TEOS) was added dropwise under vigorous magnetic stirring for 24 h to form the SiO_2_ layers. Si@SiO_2_ composites were collected by filtering and washed thoroughly with distilled water. Subsequently, a certain amount of liquid-polyacrylonitrile (LPAN) were mixed with Si@SiO_2_ composite and ground in a QM-3SP2 planetary ball mill for 10 h. The mixtures were cured in air at 220 °C for 3 h and carbonized at 1000 °C in an argon atmosphere for 5 h to form Si@SiO_2_@G composites. Finally, the Si@void@G composites were obtained by washing the products with HF to remove the SiO_2_ layers. As a comparison, bare Si nanoparticles were also coated with N-doped graphene-like sheets without SiO_2_ layer to obtain Si@G composite.

### Physical Characterization

The morphology and structure of the samples were observed by using a LEO1530 scanning electron microscope (SEM, Germany) and a Tecnai G2 transmission electron microscope (TEM, FEI, USA). The crystalline structures were obtained by a D8 advance X-ray diffraction spectrometer (XRD, Bruker, Germany) using Cu Kα radiation. Thermogravimetric analysis (TGA) results were obtained with a STA409PC TG-DSC/DTA instrument (Netzsch, Germany) from 30 to 800 °C with a heating rate of 10 °C min^−1^ in air. Raman measurements were carried out at room temperature using a Jobin Yvon/Atago Bussan T64000 triple spectrometer equipped with micro-optics. X-ray photoelectron spectroscopy (XPS) was carried out on the ESCAlab220iXL electron spectrometer from VG scientific using 300-W Al Kα radiation.

### Electrochemical Measurements

Electrochemical tests were performed by coin-type 2032 cells (Shenzhen Kejingstar Technology Co. Ltd., People’s Republic of China) which were assembled in an Ar-filled glove box (MBRAUN, Germany) with oxygen and moisture contents of less than 0.1 ppm. For preparing the working electrode, a slurry mixture of prepared active materials, carbon black, and sodium alginate in a weight ratio of 6:2:2 in water was coated on a copper (Cu) foil by an automatic film applicator (AFA-II, Shanghai Pushen Chemical Machinery Co., Ltd., People’s Republic of China). The Cu foil was dried at 70 °C for 12 h and then cut into pieces with a diameter (*ϕ*) of 14 mm. The loading of active material was ~0.42 mg cm^−2^. Subsequently, the pieces were dried at 110 °C for 6 h in vacuum. A solution of 1 M LiPF_6_ in ethylene carbonate (EC) and dimethyl carbonate (DMC) (1:1 *v*/*v*) was served as electrolyte.

The galvanostatic charge-discharge performance was assessed on a battery testing system (LAND-CT2001A, People’s Republic of China) at room temperature between cut-off potentials of 1.00 and 0.01 V at different densities. Cyclic voltammetry (CV) was recorded on an electrochemical workstation (1470E Cell Test System, Solartron, UK), with a scanning of rate of 0.1 mV s^−1^ at room temperature. Electrochemical impedance spectroscopy (EIS) was measured with a Solartron Impedance analyzer 1260A by applying an alternating current (AC) voltage of 5 mV in the frequency range from 100 kHz to 0.1 Hz.

## Results and Discussion

**Fig. 1:**
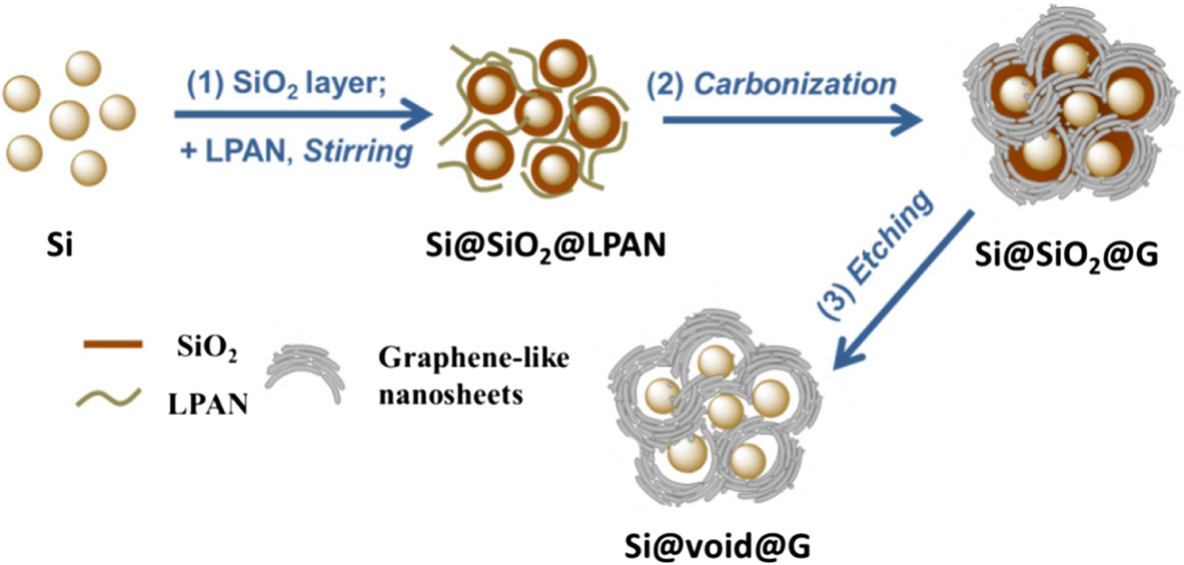
Schematic illustration of the formation route of Si@void@G composite

The design for Si@void@G composite is inspired by the structure of the tremella, where the Si nanoparticles are uniformly dispersed in the conductive and porous N-doped graphene-like nanosheets, as depicted in Fig. 1. First, a SiO_2_ sacrificial layer was formed on the surface of the Si nanoparticles. Then, a facile one-step carbon-thermal method was applied to coat Si@SiO_2_ with N-doped graphene-like nanosheets derived from a LPAN precursor. After selectively removing the SiO_2_ layer by hydrofluoric acid (HF) treatment, the tremella-like nanostructure of Si@void@G composite was obtained [15, 37, 38]. The designed 3D porous tremella structure provides a void space between Si nanoparticles and graphene-like nanosheets, leaving enough space for the expansion of Si nanoparticles. In addition, the N-doped graphene-like nanosheets provide the electrode with more flexibility as well as electrical contact to maintain good mechanical properties [37].

### Structure and Characterization

**Fig. 2:**
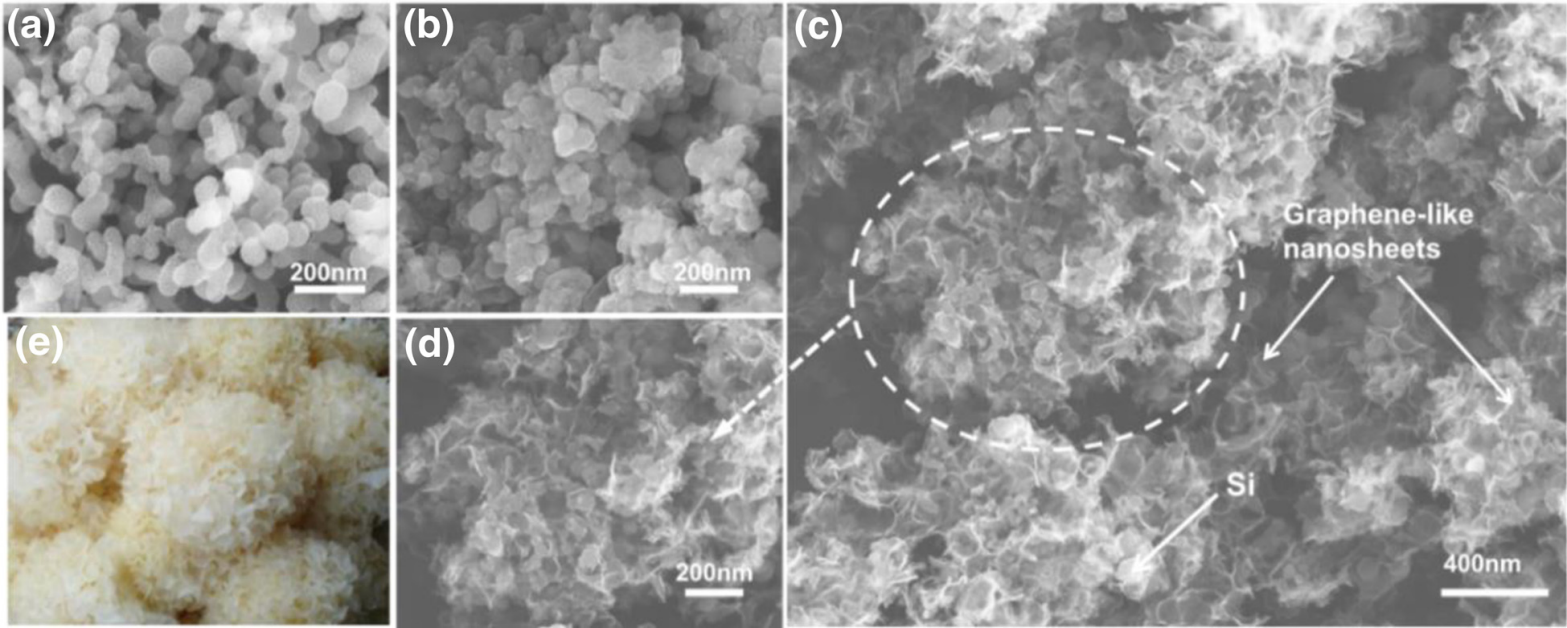
SEM images of **a** bare Si, **b** Si@G, and **c**, **d** Si@void@G. **e** Picture of tremella

Figure 2a exhibits the SEM image of bare Si nanoparticles, with the size of 60–100 nm. The SEM images of Si@G composite are presented in Fig. 2b, and the Si nanoparticles are coated with a graphene-like layers. As shown in Fig. 2c, d, the morphology of Si@void@G composite is similar to the structure of the tremella (Fig. 2e). The graphene-like nanosheets act as the tremella with a bulk of void shells, in which the Si nanoparticles are dispersed.

**Fig. 3:**
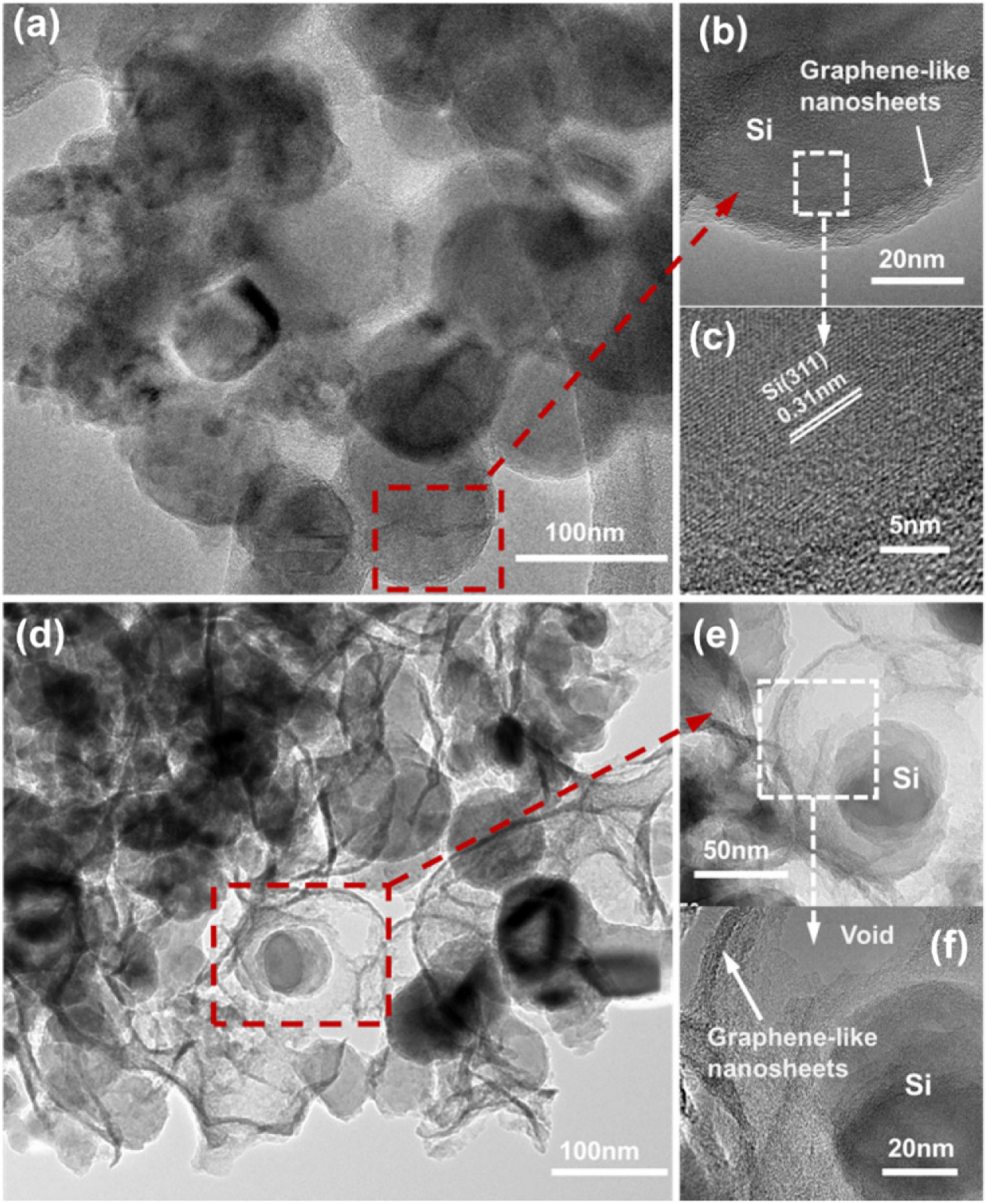
TEM images of **a**–**c** Si@G and **d**–**f** Si@void@G

The TEM images of Si@G composite are presented in Fig. 3a–c; it can be seen that the Si nanoparticles are wrapped in the graphene-like nanosheets and there are no spaces between Si nanoparticles and graphene-like nanosheets. The lattice spacing of 0.31 nm is originated from the Si (111). While for the Si@void@G composite (Fig. 3d), the Si nanoparticles are dispersed among the graphene-like nanosheets. From the high-resolution TEM images (Fig. 3e, f), void spaces between the Si nanoparticles and the graphene-like nanosheets can be observed obviously, which can provide enough room for expansion and contraction following lithiation and delithiation processes, and the thickness of graphene-like nanosheets is about 5 nm.

**Fig. 4:**
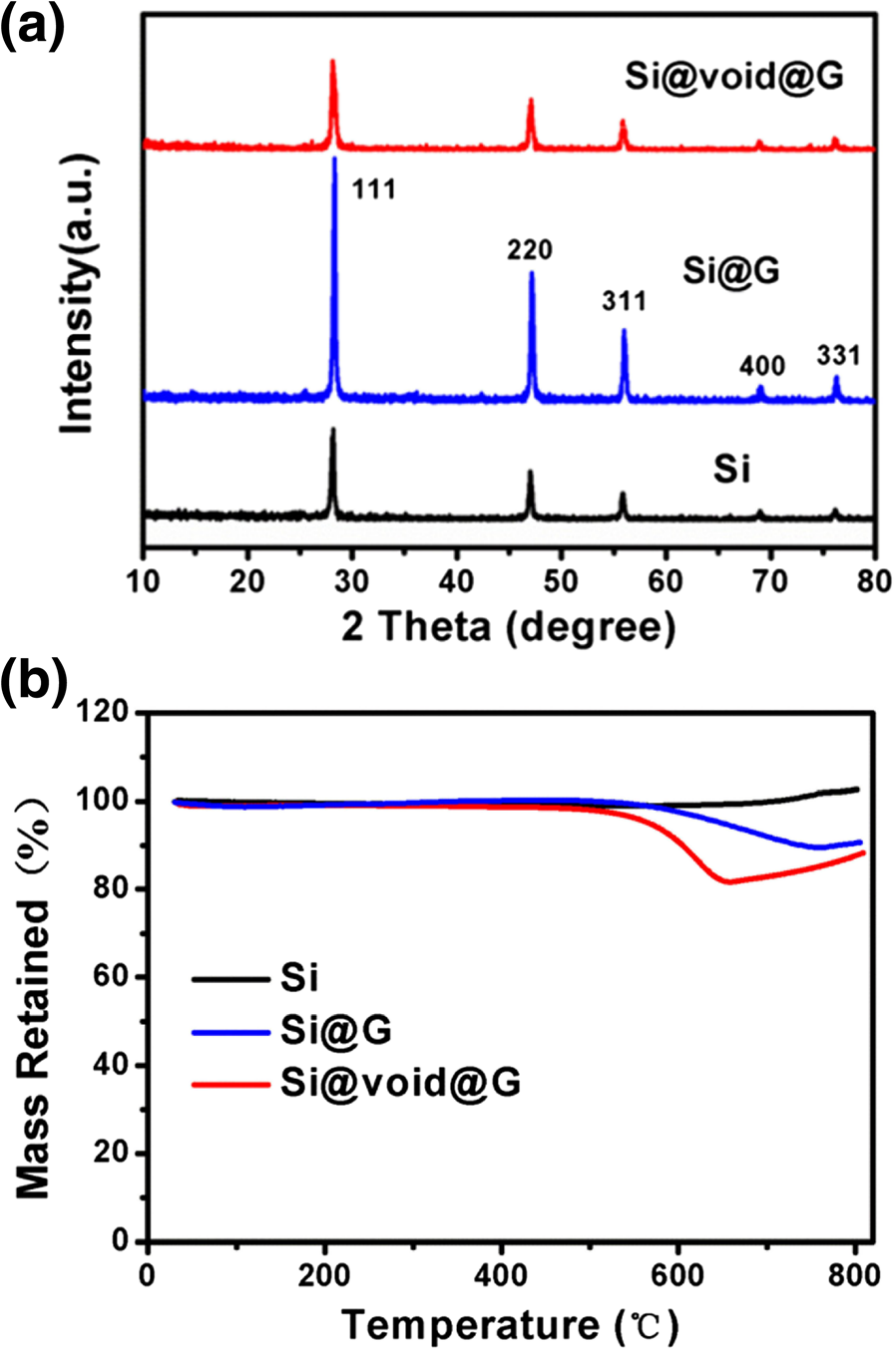
**a** XRD patterns of bare Si, Si@G, and Si@void@G. **b** TGA curves of Si, Si@G, and Si@void@G composites

XRD patterns of bare Si, Si@G, and Si@void@G composites are shown in Fig. 4a. For all the three samples, the major diffraction peaks of 28.1°, 47.0°, 55.9°, 68.9°, and 76.1° can be indexed to lattice plane of (111), (220), (311), (400), and (331) of well-crystallized silicon, respectively [39]. Compared with the diffraction peaks of bare Si, no obvious difference for the main peaks of Si@G and Si@void@G composites were found, indicating that the Si in the Si@G and Si@void@G composites retain the same crystalline structure. Figure 4b presents the TGA curves of Si, Si@G, and Si@void@G composites at 30–800 °C in air. There is a certain temperature that the graphene-like nanosheet begins to react with oxygen in air to generate CO_2_, and only Si is left alone. The oxidation of Si powder in air is not significant at 720 °C, while the oxidation reaction for graphene-like nanosheets with oxygen in air is completed at 720 °C. The weight percentages of Si in the two composites are calculated to be 89.3 % for Si@G composite and 81.7 % for Si@void@G composite.

**Fig. 5:**
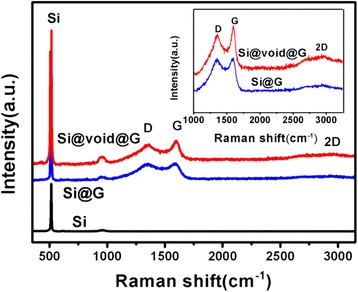
Raman spectra of bare Si, Si@G, and Si@void@G

The typical Raman spectra of bare Si, Si@G, and Si@void@G composites are exhibited in Fig. 5. The strong peak at ~513 cm^−1^ corresponds to the Raman mode of crystalline Si. Two prominent peaks observed at approximately 1355 and 1590 cm^−1^ for Si@G and Si@void@G samples correspond to the disordered bands (D bands), denoting the defects in the graphene layer, and graphitic bands (G bands), denoting the sp^2^ bond for the graphene, respectively. The broad peak at around 2600~3100 cm^−1^ represents the 2D band of carbon materials, indicating the presence of graphene in Si@G and Si@void@G composites [37]. The intensity ratio of the D band and the G band (*I*_D_/*I*_G_) reflects the degree of graphitization, defects, and the domain size of graphitization [39, 40]. The *I*_D_/*I*_G_ value for Si@G and Si@void@G composites is 0.94 and 0.91, respectively; it indicates that the composites are coupled with partial graphite and can provide better electronic conductivity.

**Fig. 6:**
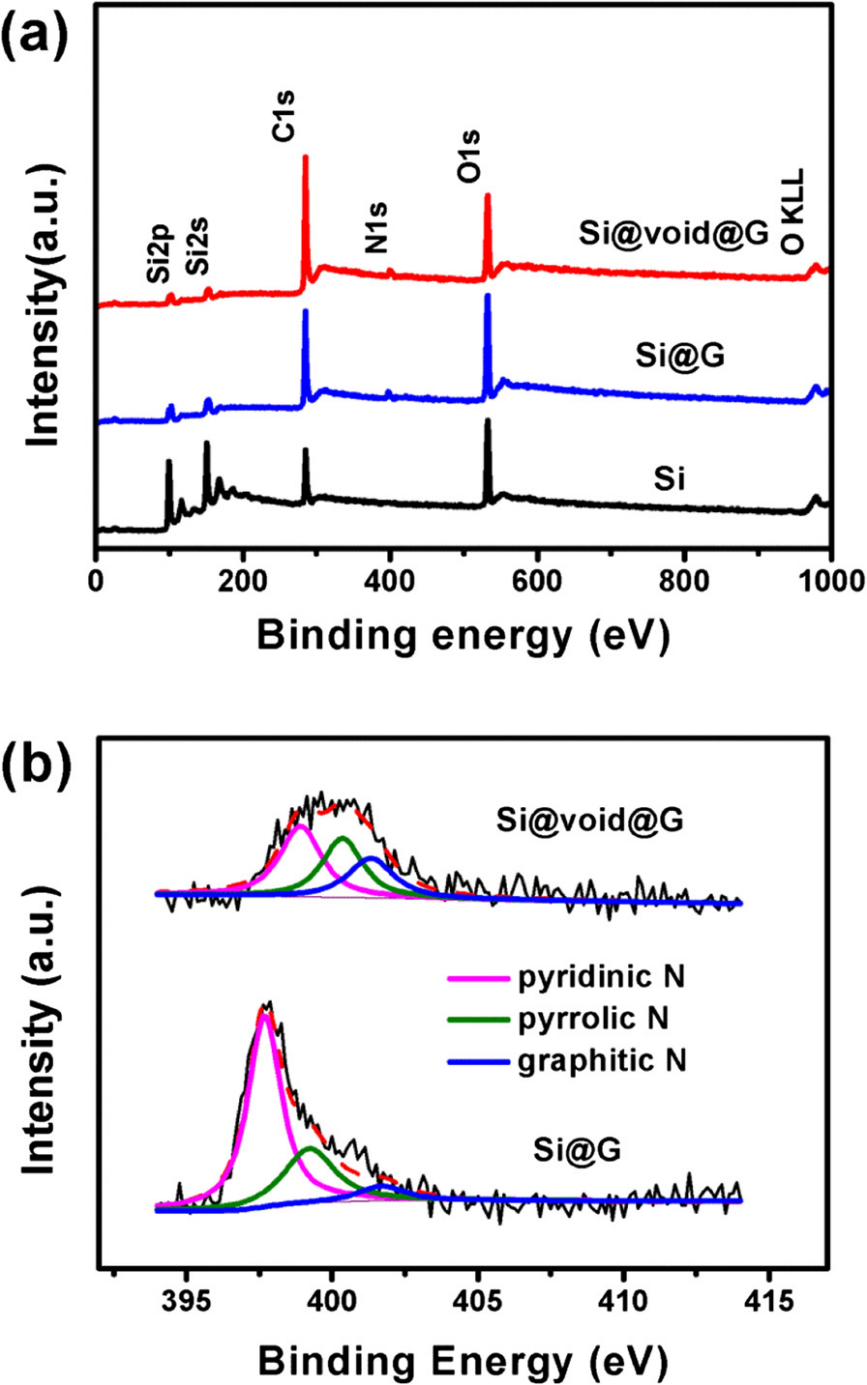
**a** XPS spectra of Si, Si@G, and Si@void@G composites. **b** XPS N1s spectra of Si@G, and Si@void@G composites

XPS was used to reveal the elemental information for bare Si, Si@G, and Si@void@G composites. Figure 6a shows that the contents of Si@G and Si@void@G composites are Si, C, O, and N, while there is no N in the bare Si. After the coating process, the intensities of the C1 s peak for Si@G and Si@void@G increase, indicating the presence of graphene-like nanosheets. The N content of Si@G and Si@void@G is 2.7 and 2.8 at.%, respectively. A detailed analysis of the high-resolution spectra of the N 1s (Fig. 6b) reveals three peaks: pyridinic N (398.2 eV), pyrrolic N (400.1 eV), and graphitic N (401.7 eV). Among these three forms of N binding, pyridinic N is being viewed as the most suitable for facilitating the electronic conductivity and the charge transfer at the interface in LIBs [37, 41]. As observed, the peak intensity for pyridinic N in Si@G and Si@void@G composites is higher than that of the other two components, contributing to the enhancement of electronic conductivity for the electrodes.

### Electrochemical Performance

**Fig. 7:**
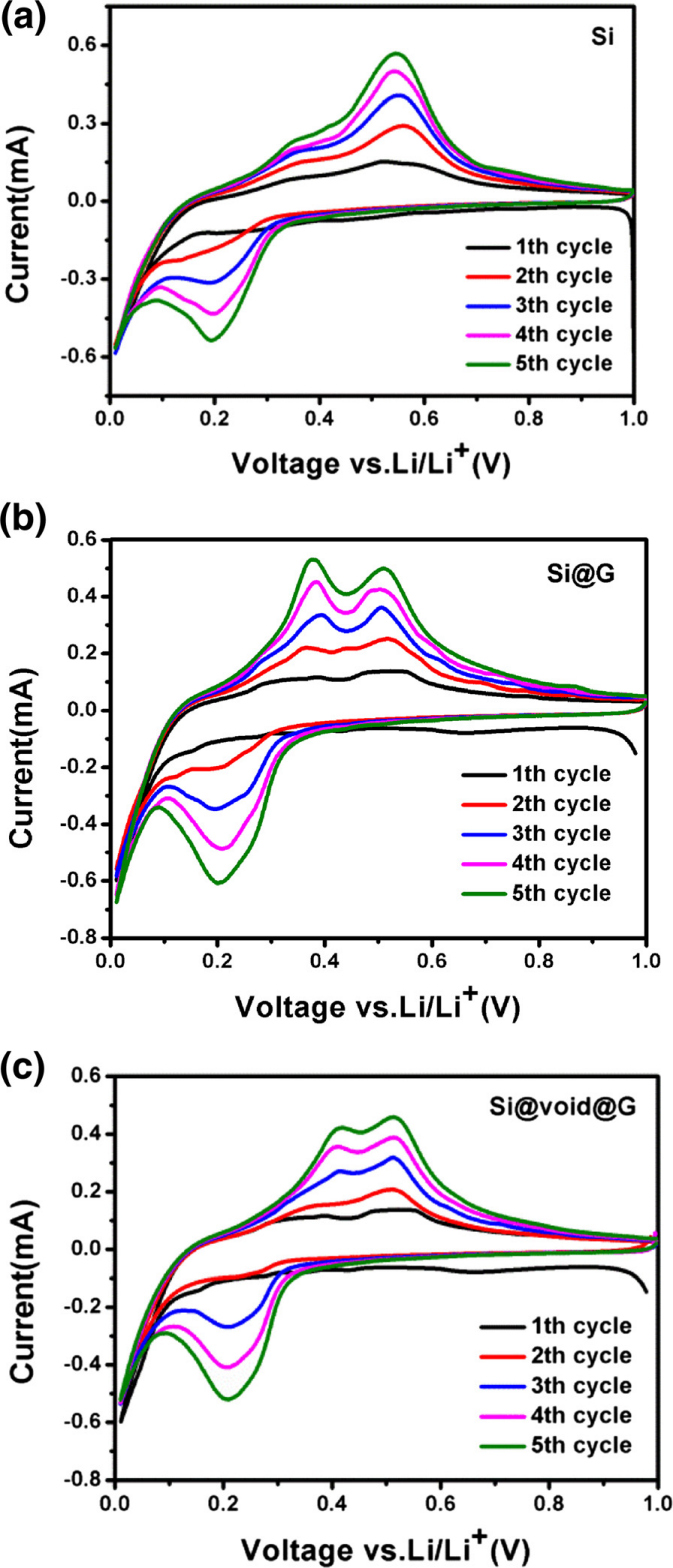
CV curves of **a** Si, **b** Si@G, and **c** Si@void@G for the first five cycles

To characterize the electrochemical performance, the first five cyclic voltammogram curves of Si, Si@G, and Si@void@G composites in a half cell in the range of 0.01–1.0 V at a scan rate of 0.1 mV s^−1^ are shown in Fig. 7. Since the process of alloy of Si is in a confined space, the Si nanoparticles may be under the irreversible shape changes upon the initial Li insertion. Then, the Si-Li alloy could exhibit reversible shape changes in subsequent cycles [42]. In the first cathode scan, there is a low reduction peak in Si@G and Si@void@G composites at approximately 0.67 V. This is attributed to the formation of a SEI film and disappears in subsequent cycles, which causes the initial irreversible capacity. After the first cycle, there is a strong reduction peak that appeared at approximately 0.20 V during discharging, due to the conversion of amorphous Si to Li_*x*_Si. Additionally, the intensities of typical anodic peaks at 0.42 and 0.51 V increase gradually in the first five cycles, suggesting the existence of probable activating processes; similar phenomena have been observed elsewhere [29, 38, 40].

**Fig. 8:**
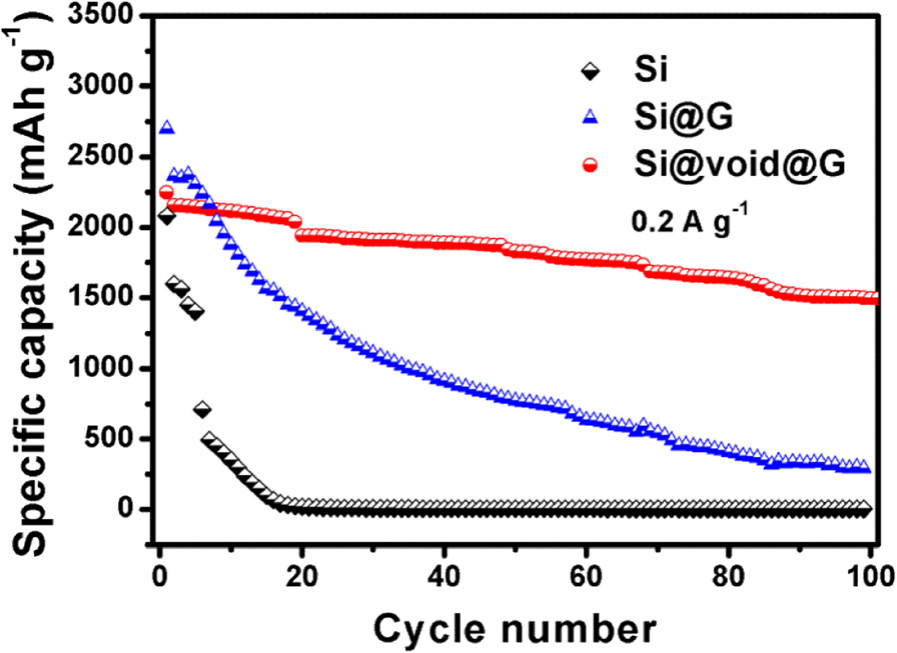
Cycling performance of bare Si, Si@G, and Si@void@G electrodes

The cycling behavior of bare Si, Si@G, and Si@void@G composites is evaluated by galvanostatic discharge-charge measurements at 0.2 A g^−1^, as shown in Fig. 8. All the three samples show a high initial discharge capacity; the bare Si nanoparticles electrode displays a rapid capacity decline from 2200 to 20 mAh g^−1^ after 20 cycles, which is because the massive volume change leads to the loss of electrical contact between the active materials and the electrode framework. After coating with conductive graphene-like nanosheets, the Si@G electrode can retain the specific capacity of 770.6 mAh g^−1^ after 50 cycles, showing a slightly improved cycling performance compared to bare Si nanoparticles, which is mainly due to the advantages of graphene-like nanosheets. And the specific capacity of tremella structure of Si@void@G composite is much higher than that of bare Si and Si@G composites. Importantly, the Si@void@G electrode displays a reversible capacity of 1497.3 mAh g^−1^ with a coulombic efficiency of 73.8 % after 100 cycles, and the capacity retention value is 66.7 %. This could be attributed to the good conductivity of graphene-like nanosheets and the fact that there is enough void space to accommodate the full expansion of Si nanoparticles.

**Fig. 9:**
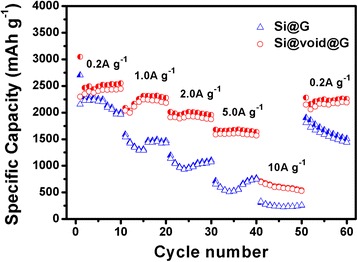
Rate capability of Si@G and Si@void@G

Figure 9 reveals the rate capability of Si@G and Si@void@G electrodes at different current densities ranging from 0.2 to 10 A g^−1^. It is noted that the capacity decreases gradually with the increase of current density. The Si@void@G electrode delivers the average discharge capacity of 2500, 2300, 2000, 1670, and 610 mAh g^−1^ at current densities of 0.2, 1.0, 2.0, 5.0, and 10 A g^−1^, respectively, and shows obvious higher discharge capacity than that of Si@G composite at a high current density. After the ultrahigh rate charge/discharge cycling of 10 A g^−1^, the capacity of Si@void@G electrode recovers to an average discharge capacity of 2230 mAh g^−1^ at 0.2 A g^−1^, exhibiting an outstanding rate capability.

**Fig. 10:**
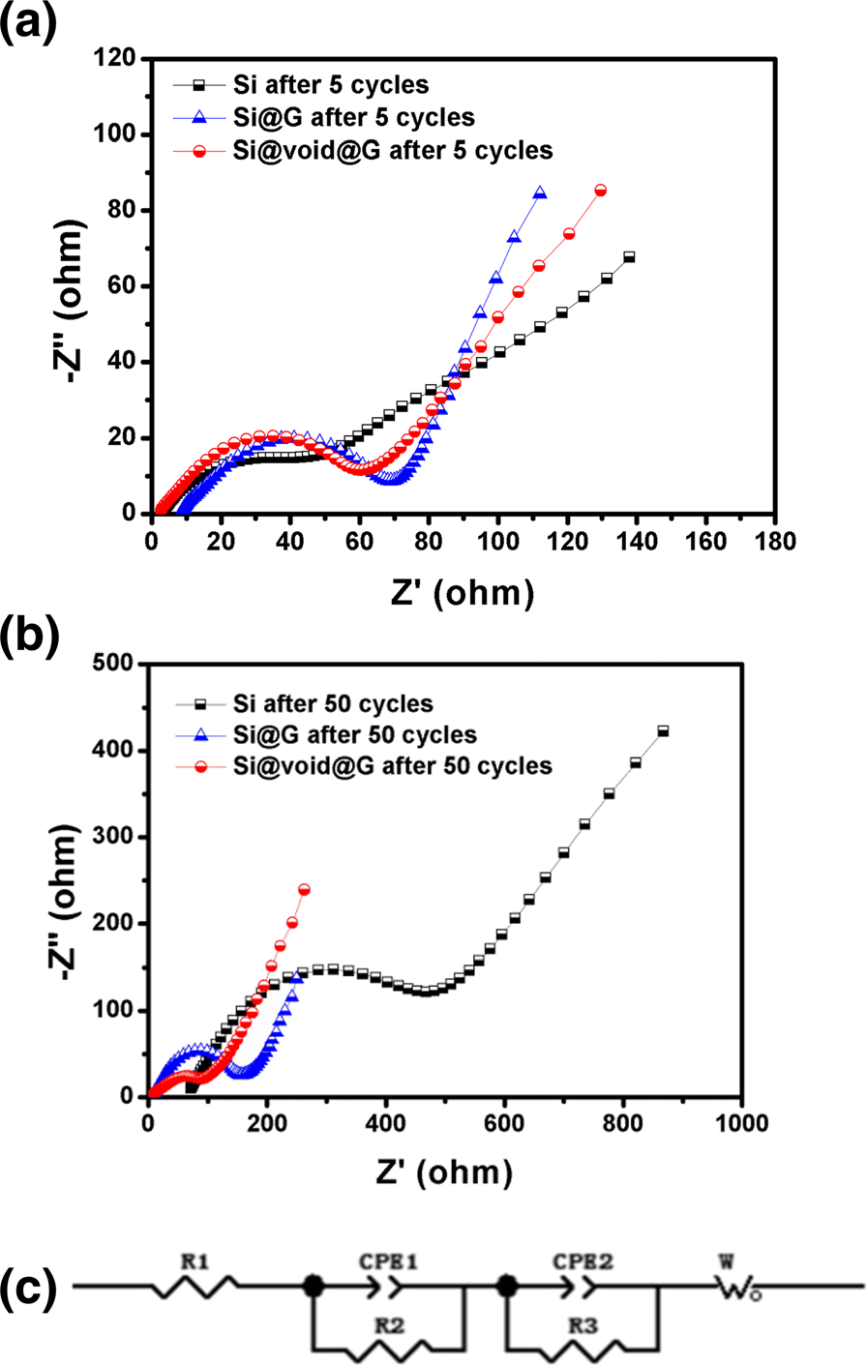
Nyquist plots of bare Si, Si@G, and Si@void@G after **a** 5 cycles and **b** 50 cycles. **c** Equivalent circuit of the EIS measurements

EIS was further examined to get insight into the stability of lithiation/delithiation of bare Si, Si@G, and Si@void@G electrodes, as shown in Fig. 10. The Nyquist plots of the three electrodes are tested in a frequency range of 100 kHz~0.1 Hz with an amplitude of 5 mV at full state of charge of 0.01~1 V, and the measured impedance data over the entire frequency range was analyzed using the equivalent circuit in Fig. 10c. The typical impedance spectra are composed of one semicircular arc at high-frequency range and a sloped straight line at low-frequency region, corresponding to the resistances of electrolyte interface and the diffusion resistance of Li-ions in the electrode materials [43, 44]. Figure 10a shows that the diameters of the semicircles for Si@G and Si@void@G electrodes after 5 cycles are smaller than that of the Si electrode, indicating lower charge-transfer resistances. Remarkably, the large semicircles for the bare Si electrode are indicative of high interfacial charge-transfer resistance because of the poor electrical conductivity of Si. No evident impedance increase was detected in Si@G and Si@void@G electrodes after 50 cycles, indicating the good conductivity and flexibility of graphene-like sheets (Fig. 10b), while the Si@void@G electrode shows smaller diameters of semicircles than that of Si@G electrode, which may be because the Si@void@G electrode can maintain the complete structure and form a stable SEI layer. The fitted impedance parameters are listed in Table 1.

**Table 1:** Kinetic parameters of Si, Si@G, and Si@void@G electrodes

Samples and cycle numbers	R1 (Ω)	R2 (Ω)	R3 (Ω)	W (Ω)
Si 5 cycles	2.543	9.703	38.92	287.5
Si 50 cycles	64.74	345.2	18.92	1846
Si@G 5 cycles	7.762	5.466	6.249	30.07
Si@G 50 cycles	8.312	116.8	47.34	421.8
Si@void@G 5 cycles	1.983	15.23	10.37	20.89
Si@void@G 50 cycles	3.576	42.9	23.86	166.9

## Conclusions

In summary, inspired by nature, a tremella structure of Si@void@G electrode with high capacity, cycling stability, and rate capacity was obtained. The Si@void@G electrode can deliver a capacity of 1497.3 mAh g^−1^ at the current density of 0.2 A g^−1^ after 100 cycles, showing better electrochemical performance than bare Si and Si@G electrodes. These results are mainly attributed to the following reasons: the unique design of tremella nanostructure provides a large void space between Si nanoparticles and graphene-like sheets to allow for the expansion and contraction of Si during the lithiation/delithiation process; the N-doped graphene-like nanosheets provide excellent electrical conductivity throughout the electrode; moreover, the Si nanoparticles are encapsulated by the thin graphene-like nanosheets, limiting the amount of SEI, which also confirms the successful design of Si@void@G materials.
